# Relative receptor affinity comparisons among inhaled/intranasal corticosteroids: perspectives on clinical relevance

**DOI:** 10.1186/1465-9921-9-75

**Published:** 2008-11-24

**Authors:** Günther Hochhaus

**Affiliations:** 1 University of Florida College of Pharmacy, Gainesville, Florida, USA

## Abstract

**Background:**

Pharmacokinetic properties, dosing regimen, and potency at the site of action are among the factors that influence activity of a corticosteroid. The potency of a corticosteroid at the site of action is determined significantly by its affinity to the glucocorticoid receptor. Recent literature on topical corticosteroids reveals an increasing emphasis on comparative relative receptor affinity values as a key method of differentiating among various corticosteroid compounds, particularly with regard to clinical efficacy.

**Methods:**

A response was formulated to: Valotis A, Högger P: Human receptor kinetics and lung tissue retention of the enhanced-affinity glucocorticoid fluticasone furoate. Respir Res 2007, 8:54.

**Results:**

Relative receptor binding affinities, while often showing significant variability across different laboratories, are a valid parameter when a comparison of the pharmacological activity of various glucocorticoids at the site of action is desired. Unfortunately within this context, scientific literature including the article from Valotis and Högger, confuse differences in potency (concentration or dose necessary to achieve a certain effect) with differences in efficacy (a quantitative difference in the overall maximum effect, even if all the receptors are occupied). All glucocorticoids will show the same efficacy as long as the selected dose will occupy the same number of receptors.

**Conclusion:**

While relative receptor affinities are useful for comparing in vitro potencies of corticosteroids, these data are not representative of physiologic conditions and should not be used as a basis for comparing the presumed effectiveness of compounds in a clinical situation.

## 

Topical corticosteroids, such as beclomethasone dipropionate, budesonide, triamcinolone acetonide, flunisolide, ciclesonide, mometasone furoate (MF), fluticasone propionate (FP), and fluticasone furoate (FF) are first-line treatments for upper respiratory allergic conditions such as asthma and seasonal/perennial rhinitis, as they provide local efficacy and, thereby, reduce systemic glucocorticoid exposure [1-4]. In recent literature, comparative relative receptor affinity (RRA) values for glucocorticoid receptors in nasal/lung tissue are increasingly used to differentiate among topical corticosteroids. In many instances, the presentation of RRA data and rank ordering of compounds is accompanied by overt or subtle implications of clinical efficacy ramifications. While RRA has a rightful place in the comparison of *in vitro *potency of corticosteroids, practicing clinicians need to understand the limitations of such data when they are used as arguments for the clinical efficacy of a given glucocorticoid.

As a case in point, I refer to a recent publication in *Respiratory Research *(2007:8:54) in which Valotis and Högger report data comparing the glucocorticoid-receptor and lung-tissue binding affinities of FF, FP, and MF [5] While the methodology of the study and the resulting data are sound, the authors imply, that a high receptor affinity, such as the one reported in this publication for FF, improves the local efficacy of a topical glucocorticoid (see Background section of the article) and that the high relative receptor affinity of the "enhanced-affinity" FF "that exceeds the RRAs of all other clinically used glucocorticoids" "may contribute to a highly efficacious profile for FF". To be fair, Högger's group is not the only one confusing potency and efficacy of inhaled glucocorticoids, as the interchangeable use of the two terms can be found throughout the literature, often with the underlying assumption that a high affinity glucocorticoid must be the better one. The clinical and pharmacologic reality, however, is that "potency" and "efficacy" are not interchangeable terms and that differences in the receptor affinity do not necessarily translate into differences in local or clinical efficacy. It only indicates that for a "weaker" glucocorticoid, higher concentrations at the site of action are necessary to achieve the same pharmacodynamic effect. In the clinical setting, differences in potency, therefore can be overcome by adjusting the dose [6]. Therefore, two glucocorticoids that differ only in the receptor binding affinity (e.g. by 25%) would show the same clinical efficacy and systemic side effects when the "weaker" steroid is given at a 25% higher dose.

In summary, while it is not my intent to discredit the importance of studying RRA, I am recommending caution in the interpretation of and reliance on such data, particularly when claims of clinical distinction are drawn. As recent pharmacokinetic/pharmacodynamic evaluations have shown, the topical selectivity and local targeting of glucocorticoids is independent of the binding affinity and solely determined by pharmacokinetic properties [7].

## Abbreviations

FF: fluticasone furoate; FP: fluticasone propionate; MF: mometasone furoate; RRA: relative receptor affinity.

## Competing interests

During the preparation of this letter to the editor, the author has been provided talks for Schering-Plough, consulted for Verus Pharmaceuticals and IVAX and received grants from AstraZeneca.

## Authors' contributions

GH is the author of this commentary.
